# Typhoid—From Past to Future

**DOI:** 10.1093/cid/ciz551

**Published:** 2019-10-15

**Authors:** Claas Kirchhelle, Andrew J Pollard, Samantha Vanderslott

**Affiliations:** 1 Wellcome Unit for the History of Medicine/Oxford Martin School, University of Oxford, United Kingdom; 2 Oxford Vaccine Group, University of Oxford, United Kingdom; 3 Oxford Vaccine Group/Oxford Martin School, University of Oxford, United Kingdom

**Keywords:** typhoid fever, interdisciplinary research, XDR typhoid, vaccination, water, sanitation, hygiene

## Abstract

*Making* *a Difference?* brings together medical humanities and sciences experts to analyze how historical and new data on typhoid control can be brought to bear on the current context of typhoid conjugate vaccine rollouts and extensively drug-resistant typhoid.

Typhoid fever is an ancient companion of humanity. Caused by the human-specific gram-negative pathogen *Salmonella enterica* serovar Typhi (*S.* Typhi) and spread by contaminated water, food, and asymptomatic carriers, typhoid has historically been presented as a scourge of armies and cities and as a killer of kings and paupers [1]. While typhoid has vanished from most high-income countries, it remains endemic in many low- and middle-income countries (LMICs). Despite the long tradition of scientific interest in typhoid, typhoid control has not attracted the same level of funding and public attention as other, more prominent diseases like the “big 3”: human immunodeficiency virus/AIDS, tuberculosis, and malaria [2, 3]. After decades of relative political neglect, extensively drug-resistant (XDR) *S.* Typhi strains and new conjugate vaccines are causing a resurgence of scientific, commercial, governmental, and nongovernmental interest in typhoid control [4].

But how should typhoid control be structured? Combining current research with an analysis of past interventions offers a way forward. In the 163 years since William Budd’s first article on waterborne typhoid transmission was published [5], numerous typhoid interventions have been implemented across the world. Not every intervention was successful, and some successful interventions may no longer be appropriate in times of spreading antimicrobial resistance (AMR) and climate change. However, others may still prove useful.

*Making a Difference?* results from a 2018 international workshop in Oxford, which brought together medical humanities and sciences experts, international donors, and policymakers to assess historical and contemporary aspects of typhoid control. The interdisciplinary workshop explored historical and current perspectives on typhoid and public health interventions, including the envisioned challenges of rolling out new typhoid conjugate vaccines and other control measures such as improved water, sanitation, and hygiene (WASH) systems.

Building on the 2018 workshop, the 6 interdisciplinary articles of this supplement highlight the potential of jointly studying the biological, medical, socioeconomic, cultural, and historical factors influencing typhoid prevalence. Vanderslott et al use historical and epidemiological methodologies to highlight the role of cheap debt in allowing British and American municipalities to curb typhoid with WASH interventions during an era of competing theories of typhoid proliferation. Harrison et al emphasize the historical role of military and colonial needs in fostering research on asymptomatic carriers and vaccine development in Germany, France, and Britain as well as the influence of different political traditions in fostering vaccine resistance or acceptance. Kirchhelle et al combine historical and genetic approaches to highlight the effects of post-1930s antibiotic use, national biosecurity agendas, and fragmented international policies in fostering a relative global neglect of typhoid and rising AMR in LMICs until the late 1990s. Pitzer et al assess current typhoid burdens and surveillance gaps in Sierra Leone, Fiji, Malawi, Vietnam, India, and Nepal and highlight the role of poverty and antibiotic overuse for XDR typhoid resurgence. Although the authors point to the need for improved epidemiological surveillance, they emphasize that there is no universal roadmap to typhoid control as well as the need to promote local ownership of research and policy decisions in endemic countries. Meiring et al discuss the ethical and practical challenges of vaccine development and rollout with regard to the new typhoid conjugate vaccine. The authors highlight the utility of mathematical efficacy modeling and of controlled human infection models for the planning of vaccine interventions. Last, Carey et al summarize current international and donor control strategies. While governmental and nongovernmental organizations are devoting more attention and resources to typhoid control, the long-term success of control measures strongly depends on generating local public acceptability.

The joint-authored articles in *Making a Difference?* make an important contribution to current debates about typhoid control and highlight the potential of using interdisciplinary approaches to study major global health challenges. While the supplement’s historical and current case studies show that there is no universal roadmap for typhoid control, robust international strategies should be multipronged, locally tailored, and pair the long-term strengthening of national LMIC surveillance and healthcare capabilities with enhanced funding for new technical interventions such as vaccines, community engagement of local populations, and the provision of affordable credit for ownership and sustainable maintenance of clean water and sanitation infrastructures at the municipal level.

## Notes

***Acknowledgments.*** The authors thank participants of the Higher Education Funding Council for England Global Challenges Research Fund (HEFCE GCRF)–funded 2018 “Typhoid: Past and Present” workshop at the University of Oxford for providing the impetus for this special supplement. They also thank the New Venture Fund for their support in financing both the publication and open access to this supplement.

***Financial support.*** The authors report grants from the Bill & Melinda Gates Foundation, Wellcome Trust, National Institute for Health Research (NIHR) Oxford Biomedical Research Centre, HEFCE GCRF, and New Venture Fund during the conduct of the study. A. J. P. is Chair of the DHSC’s JCVI and the European Medicines Agency’s Scientific Advisory Group on Vaccines. A. J. P. is also a member of the WHO’s Strategic Advisory Group of Experts on Immunization and an NIHR Senior Investigator.

***Supplement sponsorship.*** This supplement has been funded by the New Venture Fund.

***Potential conflicts of interest.*** The authors: No reported conflicts of interest. All authors have submitted the ICMJE Form for Disclosure of Potential Conflicts of Interest. Conflicts that the editors consider relevant to the content of the manuscript have been disclosed.
